# Case Report: HIT-related valve thrombosis in an LVAD patient

**DOI:** 10.3389/fcvm.2026.1820316

**Published:** 2026-06-11

**Authors:** Dan Wang, Haibo Ren, Wenjun Zhou

**Affiliations:** Department of Critical Care Medicine, Wuhan Asia Heart Hospital, Wuhan, China

**Keywords:** anticoagulation therapy, argatroban, heparin-induced thrombocytopenia, left ventricular assist device, valve thrombosis

## Abstract

This report presents a case of a patient with a history of mechanical aortic valve replacement due to aortic valve disease, who underwent left ventricular assist device (LVAD) implantation combined with concomitant redo aortic valve replacement (using a bovine pericardial bioprosthesis). Postoperatively, the patient developed unstable LVAD parameters. Subsequent imaging revealed thrombus formation on the bioprosthetic aortic valve and within the perivalvular space. The patient subsequently underwent emergency aortic valve thrombectomy, during which a white thrombus was observed. Laboratory tests confirmed the diagnosis of heparin-induced thrombocytopenia (HIT). The anticoagulation regimen was promptly switched to the direct thrombin inhibitor argatroban, after which the patient's condition stabilized. This case highlights the diagnostic and therapeutic challenges of managing HIT-related thrombosis in the setting of continuous-flow LVAD support and aims to review the underlying pathophysiology and optimal anticoagulation strategy.

## Introduction

The use of left ventricular assist devices (LVADs) has become an established therapeutic strategy for patients with end-stage heart failure (1). For those with a pre-existing mechanical aortic valve prosthesis, current guidelines recommend concomitant replacement with a bioprosthetic valve at the time of LVAD implantation to mitigate the risk of thromboembolic complications. The presence of HIT—an immune-mediated disorder triggered by heparin exposure, marked by thrombocytopenia and a pronounced predisposition to thrombosis—further complicates anticoagulation management in these patients (2). This case underscores the critical importance of maintaining a high index of suspicion for HIT in LVAD patients with unexplained thrombosis and thrombocytopenia, and the necessity for timely intervention with non-heparin anticoagulants such as argatroban.

## Case report

The patient was a 61-year-old male who underwent mechanical aortic valve replacement in 2003 for rheumatic valvular heart disease. Postoperatively, he was maintained on long-term, regular anticoagulation therapy with warfarin (2.5 mg/day), with periodic monitoring of the International normalized ratio (INR) for dose adjustment. In 2021, he was diagnosed with non-ischemic dilated cardiomyopathy complicated by ventricular tachycardia, leading to the implantation of a cardiac resynchronization therapy defibrillator (CRTD). In October 2025, he was re-admitted due to end-stage heart failure (left ventricular ejection fraction 26%), complicated by ventricular arrhythmias and recurrent ICD discharges. After evaluation by a multidisciplinary team, it was decided to proceed with LVAD implantation. Given the thrombotic risk associated with his existing mechanical aortic valve, a concomitant redo aortic valve replacement with a bioprosthesis was planned. The surgery was performed under general anesthesia, hypothermia, and cardiopulmonary bypass. It involved the implantation of an LVAD (Suzhou Tongxin CH-VAD system) and aortic valve replacement (23A bovine pericardial valve, Shanghai Cingular Biotechnology). Intraoperative transesophageal echocardiography (TEE) assessment showed: the LVAD inflow cannula was oriented towards the mitral orifice and parallel to the interventricular septum; the newly replaced aortic valve functioned well with no paravalvular leak detected. The procedure was successful, and the patient was transferred to the cardiac intensive care unit (ICU) postoperatively.

**Figure 1:**
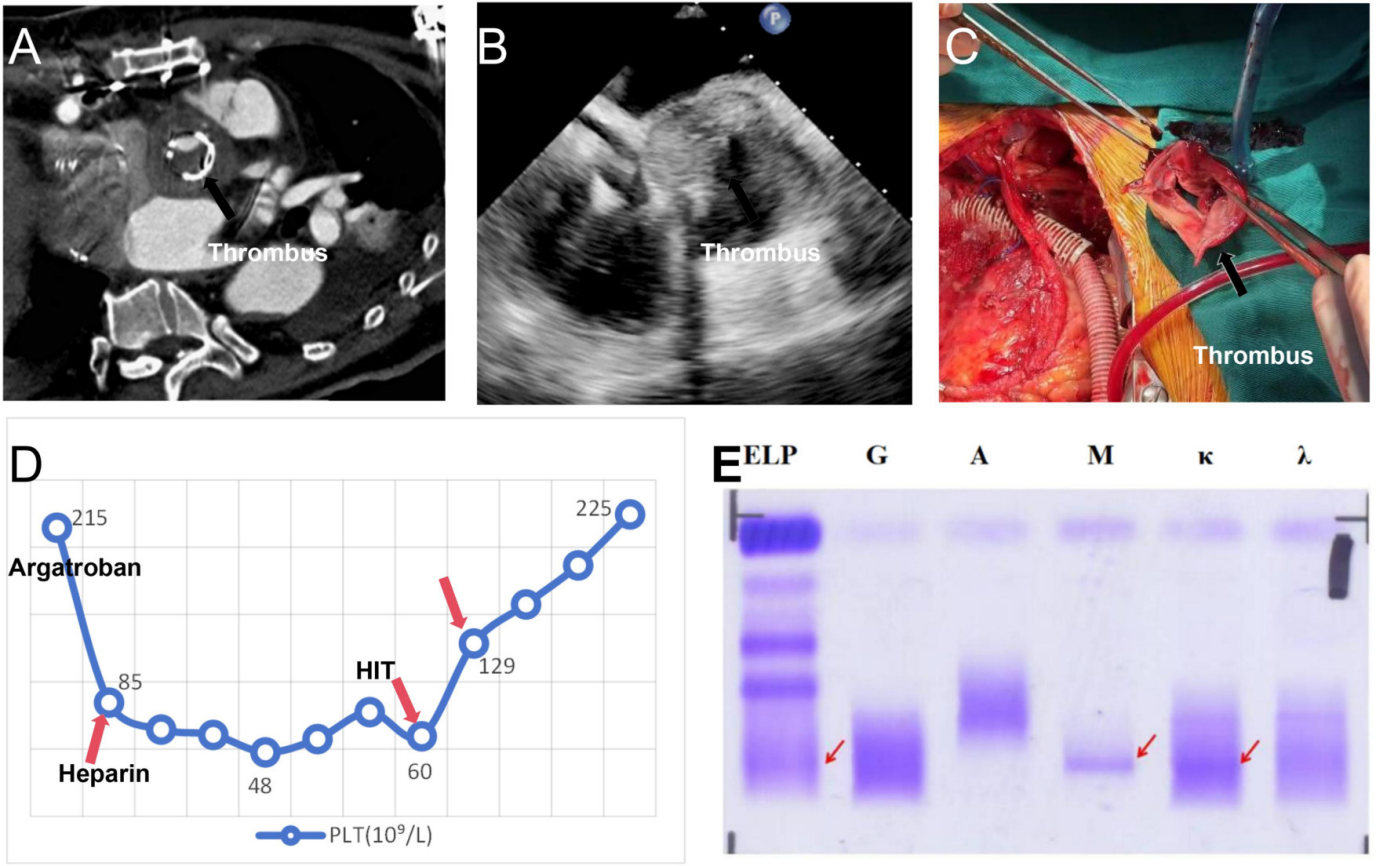
Imaging and laboratory findings of HIT with aortic valve thrombosis. **(A)** CTA demonstrates thrombus formation on the bioprosthetic aortic valve (black arrowhead). **(B)** The TEE shows thrombus formation on the bioprosthetic aortic valve (black arrowhead). **(C)** Intraoperatively retrieved white thrombus (black arrowhead). **(D)** Time-dependent changes in platelet count following heparin exposure and argatroban initiation. **(E)** Serum Immunofixation Electrophoresis. The electrophoretogram demonstrates an IgM-κ type monoclonal immunoglobulin. Monoclonal immunoglobulin band (indicated by the red arrowhead).

The initial postoperative anticoagulation regimen consisted of intravenous heparin, later bridged to oral warfarin with a target INR range of 2.0–3.0 or an aPTT of 60.0–70.0 s. On postoperative day 5, LVAD monitoring revealed a set speed of 2,800 RPM with flow fluctuations and a power of 2.8 W. Transthoracic echocardiography images were of poor quality and no thrombus was detected. The following day, flow remained persistently low at 1.5–2.0 LPM, and increasing the speed to 3,200 RPM did not significantly improve flow. The patient then developed chest tightness and progressive dyspnea. Laboratory studies indicated a progressive decline in platelet count from 215 × 10⁹/L preoperatively to 60 × 10⁹/L (Figure 1D), along with elevated D-dimer and thrombin–antithrombin III complex (TAT) levels. CTA imaging (Figure 1A) demonstrated the absence of aortic valve opening, with no contrast transit between the ascending aorta and left ventricle. A thrombus was suggested by hypodense areas around the valve annulus and subvalvular region, accompanied by wall thickening of the aortic sinus and proximal ascending aorta (maximal thickness ∼13 mm). TEE imaging (Figure 1B) demonstrated an approximately 15 mm × 10 mm hypoechoic mass in the aortic valve region.

Emergency thrombectomy was performed, revealing white thrombus adherent to the aortic root, annulus, and subvalvular areas (Figure 1C), which was completely removed. The valve leaflets were structurally intact and were therefore preserved, with no need for repeat valve replacement. The patient had previously received heparin but did not have HIT. Given the formation of an acute thrombus despite anticoagulation and the presence of severe thrombocytopenia, HIT was highly suspected. Subsequent evaluation confirmed the diagnosis: the 4Ts risk score was 6 (high risk), the HIT antibody test was positive at 8.0 U/mL, and immunofixation electrophoresis revealed a monoclonal IgM protein (Figure 1E). Following the discontinuation of heparin and initiation of argatroban, the patient's platelet count rapidly recovered to 129 × 10⁹/L (Figure 1D), with a marked decrease in coagulation activation markers (including D-dimer and TAT), resolution of clinical symptoms, and stabilization of LVAD flow. The patient was successfully transitioned to a long-term oral anticoagulation regimen of warfarin (INR target 2.0–3.0) combined with aspirin 100 mg/day. After achieving clinical stability, the patient was transferred out of the ICU and scheduled for regular follow-up. The patient was followed up for 6 months after discharge and recovered well.

## Discussion

This patient, who developed HIT-related thrombosis following LVAD implantation and aortic valve replacement, presented multifaceted anticoagulation challenges in clinical management. These challenges stemmed from the synergistic effects of the following high-risk factors: the inherent thrombogenicity of the LVAD itself, the thrombogenicity of the prosthetic bioprosthetic valve, and the intense immune-mediated procoagulant state triggered by HIT.

### Hemodynamic basis of thrombus formation

In patients undergoing combined LVAD implantation and aortic valve replacement, postoperative thrombus formation is a key challenge in clinical management. While aortic valve replacement anatomically preserves the valve's opening and closing functions, the continuous flow generated by LVAD significantly reduces left ventricular pulsatility. Concurrently, left ventricular unloading leads to decreased antegrade flow across the aortic valve, resulting in reduced aortic leaflet motion and insufficient aortic valve opening, or even a persistently closed state. This haemodynamic alteration predisposes the aortic root to blood stasis, thereby promoting thrombus formation (3). Analysis of the IMACS international multicenter registry (*n* = 15,267) showed that the incidence of thromboembolism was 8% in patients undergoing concomitant aortic valve surgery during LVAD implantation, which was not statistically different from the 9% incidence in those without aortic valve surgery (4). Whether allowing intermittent aortic valve opening can reduce the risk of thrombosis requires further research. Furthermore, optimising device speed control algorithms to promote periodic aortic valve opening while achieving adequate left ventricular unloading is also a critical issue requiring precise balancing in current management strategies.

Furthermore, compared to porcine pericardial valves, bovine pericardial valves generally offer a larger effective orifice area and superior hemodynamic performance, and may reduce the risk of reoperation (5). However, it must be recognized that although bioprosthetic valves inherently carry a lower thrombosis risk than mechanical valves, under the continuous impact of the high-flow, non-physiological blood flow generated by the LVAD, the flow patterns around the valve are still prone to disturbance, and their potential risk for thrombus formation remains non-negligible (6).

### Diagnostic insights and management strategy for HIT

HIT was traditionally regarded as an immune reaction mediated by polyclonal antibodies. HIT was traditionally regarded as an immune reaction mediated by polyclonal antibodies. Notably, recent studies have provided new insights into the pathogenic antibody mechanism of HIT. In particular, a study published in the New England Journal of Medicine using mass spectrometry and immunofixation electrophoresis found that all 9 patients with HIT had monoclonal anti-PF4/heparin antibodies, and 67% of the patients had detectable M protein by immunofixation electrophoresis. Functional assays further confirmed that only the monoclonal antibody component was pathogenic. This finding suggests that the pathogenic antibodies in HIT are essentially monoclonal rather than polyclonal, offering a completely new perspective on the pathogenesis of the disease (7). The high-sensitivity latex-enhanced immunoturbidimetric assay (LIA) has a core value in efficiently ruling out HIT, with a negative predictive value as high as 99.7%. A negative result essentially excludes the diagnosis and helps avoid unnecessary anticoagulation therapy. When combined with a high clinical probability (e.g., a high-risk 4Ts score), the positive predictive value of the test is significantly increased (8). However, when a patient has a concomitant monoclonal gammopathy, serological testing may encounter unique interference. It has been reported in the literature that in patients with monoclonal gammopathy of undetermined significance, immunological testing for PF4/heparin can yield false-positive results, whereas functional assays (such as SRA or HIPA) are usually negative because they are less susceptible to interference from such non-specific antibodies, thereby ruling out HIT (9). Therefore, in clinical practice, the 4Ts score should first be used to assess the clinical probability. Patients with intermediate or high clinical probability should undergo immunological testing for anti-PF4/heparin antibodies. For those with positive results or a high degree of clinical suspicion, functional testing should be further performed to confirm the diagnosis. In the present case, the patient presented with progressive thrombocytopenia and a recent history of heparin exposure, with a high-risk 4Ts score, a positive anti-PF4/heparin antibody LIA test, and detection of an IgM-κ monoclonal immunoglobulin by immunofixation electrophoresis. These findings provide key clues for the diagnosis of HIT.

For high-risk HIT, immediate discontinuation of all heparin-based agents and initiation of non-heparin anticoagulation are essential (10). Argatroban, as a direct thrombin inhibitor, effectively blocks the hypercoagulable state associated with HIT, reduces the risk of thrombotic events, and does not increase bleeding tendency (11). Furthermore, for LVAD patients, some of whom have concomitant multi-organ dysfunction, argatroban is an effective and safe medication for treating HIT, particularly suitable for critically ill patients with renal impairment (12). However, whether heparin can be reintroduced after HIT antibodies become negative remains undetermined, and most clinical centers tend to avoid its long-term use (13). According to the 2024 ISHLT consensus statement, patients with an LVAD should receive dual antithrombotic therapy with warfarin (target INR 2.0–3.0) and aspirin (81–325 mg/day) (14). For patients with an LVAD and a mechanical aortic valve replacement, persistent aortic valve closure is a feature associated with a high risk of thrombosis; lifelong therapy with warfarin and aspirin is recommended unless contraindicated, for example by active major bleeding. Although the warfarin-plus-aspirin regimen used in this case was successful, its benefit-to-risk ratio still requires further validation through prospective studies and larger sample sizes.

## Data Availability

The original contributions presented in the study are included in the article/Supplementary Material, further inquiries can be directed to the corresponding author.
